# Bioactivity and in Silico Insights of Collagen-Derived Peptides from Jellyfish (*Stomolophus* sp. 2) Mesoglea

**DOI:** 10.3390/md23110427

**Published:** 2025-11-05

**Authors:** Blanca del Sol Villalba-Urquidy, Wilfrido Torres-Arreola, Isabel Medina, Laura Estefany Hernández-Aguirre, Jesús Enrique Chan-Higuera, Josafat Marina Ezquerra-Brauer

**Affiliations:** 1 Department of Food Research and Graduate Studies, University of Sonora, Blvd. Luis Encinas y Rosales s/n, Col Centro, C.P., Hermosillo 83000, Son, Mexico; solvillalba.10@hotmail.com (B.d.S.V.-U.); laura.hernandez@unison.mx (L.E.H.-A.); enrique.chan@unison.mx (J.E.C.-H.); 2 Department of Food Technology, Marine Research Institute (CSIC), c/E. Cabello, 6, 36208 Vigo, Spain; medina@iim.csic.es

**Keywords:** bioactive peptides, in silico analysis, jellyfish hydrolysates, genotoxicity, ultrafiltration, blue cannonball jellyfish

## Abstract

Jellyfish, a promising source of bioactive compounds, has attracted the attention of the biotechnology sector. This research explored the antioxidant and antimutagenic properties and the genotoxicity of peptides derived from blue cannonball jellyfish (*Stomolophus* sp. 2) collagen hydrolysates (JCH) as potential food supplements. Firstly, JCH was fractionated into three parts based on molecular weight. Notably, the low-molecular-weight hydrolyzed fraction (<3 kDa) exhibited the highest bioactivity, with ABTS scavenging activity of 8993 ± 5.2 μmol TE/g and an antimutagenic inhibition rate against AFB_1_ of 88%. This fraction remained non-genotoxic at 100 ppm, suggesting its suitability for potential applications without evidence of genotoxic damage. In addition, in silico analysis revealed 15 unique peptides in *Stomolophus* sp. 2 collagen hydrolysates, ten of which showed particularly promising bioactive potential. Peptides from *Stomolophus* sp. 2 with molecular weights under 3 kDa exhibit remarkable bioactivity and hold great promise for future research on molecular characterization and bioactive food supplements.

## 1. Introduction

The rapid worldwide increase in jellyfish has led to a highly advantageous, unconventional source of fishery for traditional fishermen, particularly with the decay of conventional fishing resources [1]. This decay has considerably affected the traditional fishermen’s financial stability, forcing them to face severe economic difficulties [2]. Conversely, jellyfish fisheries represent a substantial opportunity for improving the economic conditions of these fishermen [3], especially with the production of bioactive peptides derived from jellyfish collagens [4].

According to systematic studies of jellyfish species, the valid species within the jellyfish family on the American continent are *Stomolophus meleagris* [5] and *Stomolophus fritillaria* [6]. In the southern Gulf of California, specifically in the Upper Gulf of California, there resides an as-yet-undefined species of *Stomolophus* jellyfish, known as *Stomolophus* sp. 2, commonly referred to as the blue cannonball jellyfish [7]. *Stomolophus* sp. 2 offers considerable profit potential for those highly dependent on fishing [3]. Moreover, jellyfish harvesting has become an economic alternative for coastal fishermen in central and southern Sonora, with about 57,000 tons reported in 2024 [8]. Their extracted collagen shows notable antioxidant capacity [9]. Its gelatin has been shown to possess antimutagenic activity [10], further supporting the potential health benefits of jellyfish collagen hydrolysates.

Jellyfish collagen hydrolysates hold significant promise as novel sources of bioactive peptides, offering a range of health benefits, including antioxidant, antihypertensive, antimicrobial, and antiproliferative effects [11]. Previous studies on marine-derived antioxidant peptides have reported remarkable free radical-scavenging and cytoprotective activities. These include investigations on peptides from redlip croaker scales [12], mussel hydrolysates [13], tuna scale gelatin [14], Siberian sturgeon cartilage [15], and other marine by-products with demonstrated bioactivity and structural diversity [16]. These antioxidant peptides, typically composed of 2 to 20 amino acids and with molecular weights below 3000 Da, have attracted increasing scientific interest [17]. Conversely, jellyfish enzymatic hydrolysis produces peptides with molecular weights lower than 8000 Da [17,18,19]. The molecular weight of marine protein hydrolysates is a crucial factor in producing bioactive peptides [20].

Membrane separation is an alternative method for obtaining peptides with specific molecular weights, widely used in industry due to its low cost, rapid processing, and high efficiency [21]. The ultrafiltration membrane separation process has been shown to enhance the specific bioactivity of jellyfish hydrolysates [22,23,24]. However, despite these research efforts, there is a significant lack of information about the application of ultrafiltration to improve the antioxidant and antimutagenic properties of *Stomolophus* sp. 2 mesoglea collagen hydrolysates. This knowledge gap underscores the significance and innovative nature of the present work.

The present work considers the mesoglea of blue cannonball jellyfish (*Stomolophus* sp. 2) as a source of collagen. It aims to document the bioactivity (antioxidant and antimutagenic) and genotoxicity of the fractions obtained by ultrafiltration after enzymatic hydrolysis of jellyfish collagen with Alcalase. Additionally, bioactive peptides encoded in the collagen hydrolysate proteomes of *Stomolophus* sp. 2 mesoglea were predicted in silico, providing molecular insight into their potential functional roles.

## 2. Results

### 2.1. Collagen and Hydrolysates: Yield and Properties

**Figure 1:**
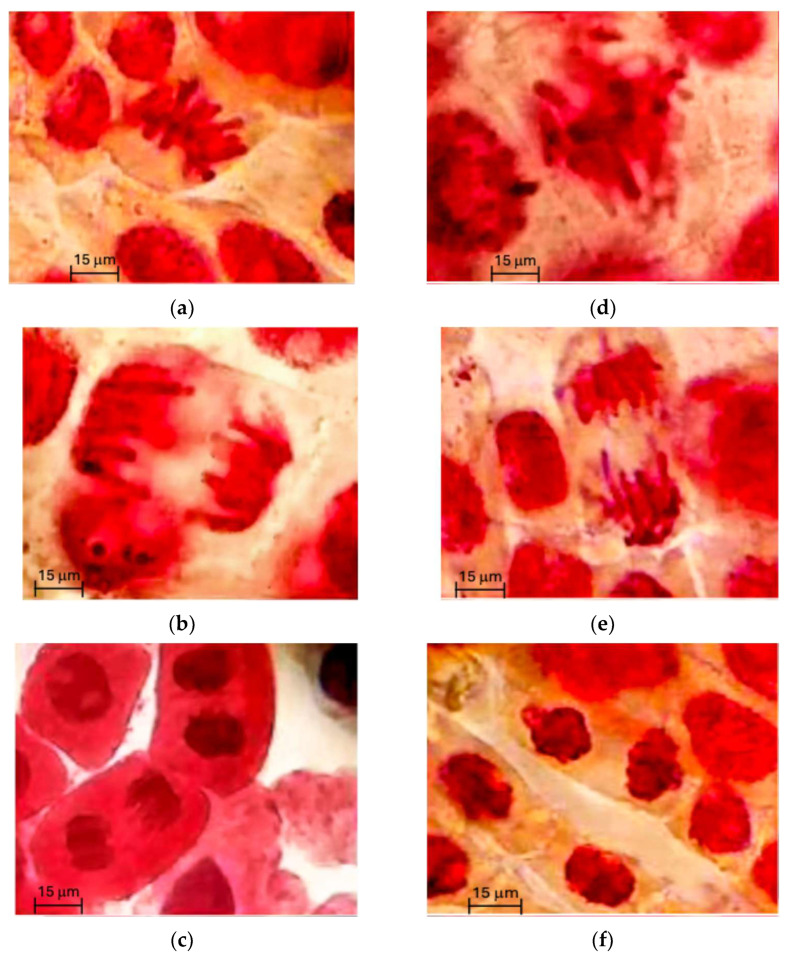
Root sections of Cambray onion (*Allium fistulosum*) observed under a light microscope (the burgundy color indicates the location of the chromosomes in the cell nucleus; the scale bar denotes 15 μm length): (**a**) normal metaphase; (**b**) normal anaphase; (**c**) normal telophase; (**d**) metaphase with chromosome breakage; (**e**) anaphase with chromosome breakage; (**f**) telophase with chromosome breakage. Negative control: water; positive control: sodium azide. Chromosomal structures were observed under a light microscope with a 100× objective lens, providing detailed visualization of their morphology.

The percentage of collagen after extraction, estimated from its hydroxyproline content (5.7 ± 0.38 g/100 g), was 60.3 ± 2.2% with a degree of hydrolysis (DH) value of 27.2 ± 1.3%. The hydrolysate yield (expressed as grams of dry hydrolysates per 100 g of collagen) was 11.08%. These hydrolysates at 100 ppm show a low level of adverse effect on chromosomes (3.4% abnormalities). Different phases of normal and abnormal mitosis are shown in Figure 1. Based on these images, it was determined whether the hydrolysates or fraction three (F3) samples exerted harmful effects on the chromosomes, as discussed later.

Antioxidant values (Table 1) showed that the obtained hydrolysates exhibited the ability to undergo single-electron transfer (SET) mechanisms against the ABTS radical, hydrogen atom transfer (HAT) capacity against the AAPH radical, and ferric reducing antioxidant power (FRAP). Moreover, these hydrolysates effectively inhibited the AFB1-induced mutation in the *Salmonella typhimurium* strain TA100, with inhibition percentages greater than 50% (Table 2), indicating moderate inhibition of the control mutagen [25].

### 2.2. Fractions Characterization

#### 2.2.1. Antioxidant Capacity

The ultrafiltration membrane used in this work, with varying molecular weight cut-offs, was used to obtain the following fractions: F1 (>10 kDa), F2 (3–10 kDa), and F3 (<3 kDa). The three obtained hydrolysate fractions showed the capacity to donate hydrogen atoms and electrons, reduce Fe^3+^ to Fe^2+^, and scavenge oxygen-derived radicals (Table 1). Among them, the most significant antioxidant activity (*p* < 0.05) was detected in fraction F3. The antioxidant activity of the samples showed clear variations among the different fractions and assays. In all methods evaluated (ABTS, FRAP, and ORAC), the fraction F3 exhibited the highest antioxidant activity, reaching 8993 µmol TE/g for ABTS, 7622 µmol TE/g for FRAP, and 599 µmol TE/g for ORAC. In contrast, the fraction F1 consistently showed the lowest values: 5763, 1244, and 223 µmol TE/g, respectively. Additionally, in the ABTS assay, the IC_50_ antioxidant capacity of the fractions confirmed that fraction F3 had the highest antioxidant capacity. The IC_50_ values were ranked as follows: F3 (0.24 mg/mL) < F2 (0.31 mg/mL) < F1 (0.42 mg/mL).

The crude hydrolysates and fraction F2 displayed moderate activity levels, positioned between F1 and F3. Specifically, the hydrolysates showed higher antioxidant capacity than F2 in the ABTS and FRAP assays but slightly lower ORAC values compared to F3. A progressive increase in antioxidant potential with decreasing membrane molecular weight cut-offs was observed across all assays, indicating a general inverse relationship between fraction size and antioxidant capacity. As expected, fractions obtained with an ultrafiltration membrane with low-molecular-weight cut-offs exhibit higher antioxidant activity [26].

#### 2.2.2. Antimutagenic Capacity

The highest antimutagenic activity, measured as the percentage inhibition of *S. typhimurium* TA100 revertants/plate, was observed in F3 even at the lowest concentration (Table 2), consistent with the antioxidant activity observed. Therefore, the peptides present in the obtained fractions may protect against the type of mutation induced by the TA100 strain, including base substitutions and frameshift mutations [27].

#### 2.2.3. Genotoxicity of Hydrolysates and Fraction < 3 kDa

The JCH and F3 did not alter the frequencies of different cell stages, and their treatments did not induce a wide range of mitotic abnormalities compared with sodium azide in the root tips of *Allium fistulosum* (Figure 1). The mitotic index (MI) of *A. fistulosum* root was not significantly (*p* > 0.05) decreased by either JCH or F3 (Table 3). The MI values and abnormality levels indicated that both hydrolysates and F3 are not genotoxic at 50 ppm or 100 ppm [28].

#### 2.2.4. Amino Acid Content of Jellyfish Hydrolysates

The hydrolysates obtained were rich in glycine, glutamic acid, arginine, aspartic acid, and proline, accounting for 23, 13, 10, 9, and 9% of the total amino acid composition, respectively. The amino acids with hydrophobic side chains—glycine, alanine, valine, leucine, isoleucine, proline, phenylalanine, and methionine—represent about 49% of the jellyfish hydrolysates (Table 4). The total essential amino acid (EAA) content is 30.6 g/100 g of protein. In comparison, the non-essential amino acid (NEAA) content is 69.8 g/100 g of protein, and the resulting EAA/NEAA ratio is 0.438 [29].

#### 2.2.5. Bioactive Peptides Identified by Informatics Analysis

The proteins identified from jellyfish protein extracts using nano-LC-MS/MS were derived from 3086 identified spectra (PSM), which included 707 distinct peptides corresponding to 10 proteins. Notably, collagen was identified among the proteins. The collagen characterized in this study was type IV, encompassing 15 unique peptides (Table 5).

Bioactive peptides remain inactive when embedded in proteins and exhibit their activity only after being released by enzymatic action. In this study, the bioactive peptides in the collagenous extracts of *Stomolophus* sp. 2 mesoglea were predicted using Peptide Ranker software (http://distilldeep.ucd.ie/PeptideRanker; accessed on 19 June 2024). The analysis revealed that 100% of the identified peptides had a molecular weight below 3000 Da, with 80% falling within the range of 1000–2215 Da and 20% below 1000 Da. Among the 15 unique peptides identified in the collagen of *Stomolophus* sp. 2 through bioinformatic analysis, ten peptides scored above 0.5 [30,31], indicating a high probability of bioactivity (Table 5).

**Table 5:** Jellyfish hydrolysate collagen peptides identified by bioinformatic analysis (accession number tr|V9GWB0|V9GWB0_CRASO ^1^).

Peptide Identified	Average Mass ^2^	Ppm ^3^	Length ^4^	Bioactive Probability ^5^
KGNEGPPGEKGL	1181	6.9	12	0.56
KGQPGPGGSADF	1116	6.9	12	0.62
PGQNGLRGADGIKGEPGL	1734	9.6	18	0.63
KGAVGEPGPKGDL	1412	7.4	14	0.62
KGEPGESGGL	929	0.4	10	0.56
GDTGLDGEKGNKGEPGARGEI	2199	9.5	22	0.47
KGDAGTNGL	831	6.3	9	0.42
AGVEGPPGPPGF	1080	6.1	12	0.80
GPPGDQGPQGL	1219	0.7	12	0.84
GSQGPTGEKGANGLPGL	1708	1.1	18	0.78
PPGDQGPQGL	964	6.6	10	0.77
GNAGPKGEPGESGGL	1495	2.3	16	0.63
PRGDPGQKGEPGQ	1420	5.2	14	0.25
KGARGLNGTGGEKGSRGPRGF	2215	6.4	22	0.50
GRDGAGVKGNAGPKGEPGESGGL	2179	3.9	24	0.36

^1^ Accession number according to PEAK STUDIO 8.5 database. ^2^ Theoretical masses of identified peptides retrieved from database. ^3^ Absolute mass error; for values ≤ 10 ppm, the potential for false positives is considered low [32]. ^4^ Theoretical lengths of identified peptides retrieved from database. ^5^ Scale: 0.0: highly unlikely to be bioactive; >0.5: high probability of bioactivity; 1.0: highly likely to be bioactive [25,26].

## 3. Discussion

It is considered that hydroxyproline content is the best means for estimating the percentage of collagen. In the present work, the collagen content detected in *Stomolophus* sp. 2 was comparable to values previously reported for other jellyfish species, such as *Rhopilema esculentum* (65%) [33] and *Rhopilema pulmo* (61.15%) [34].

The in vitro Ames antimutagenic assay is a biological assay that uses a mutant strain of *Salmonella typhimurium* to evaluate whether a substance can prevent or reduce the mutagenicity induced by a mutagenic agent. Since cancer is often linked to DNA damage, the test also serves as a rapid assay to estimate the anticarcinogenic potential of a compound. If the compound is antimutagenic, it reduces the frequency of mutations (revertants) observed in the bacteria compared to the control, indicating that the substance possesses protective properties against genetic damage [35]. The Ames test showed that the *Stomolopus* sp. 2’s hydrolysate contained peptides with antimutagenic effects, capable of inhibiting mutations or DNA damage associated with carcinogenesis. These peptides present in the hydrolysates could act as antioxidants and cell regulators, making them a promising alternative for disease prevention. However, more studies are required to confirm this.

The absence of significant chromosomal abnormalities and the maintenance of the mitotic index at both 50 and 100 ppm suggest that neither JCH nor F3 is genotoxic under the tested conditions. These findings are consistent with previous reports describing the low cytogenetic toxicity of collagen-derived peptides, reinforcing their potential for applications in food and biomedical formulations [36].

The antioxidant and antimutagenic activities detected in the obtained hydrolysates could be attributed to the presence of some amino acids, such as glycine and proline. Glycine acts as an indirect antioxidant by being a component of glutathione, a potent antioxidant in the body. It also has antimutagenic properties by suppressing the formation of free radicals and stabilizing cell membranes, thereby helping to protect against cellular damage and oxidative stress [37]. Proline, on the other hand, can act as a compatible osmolyte to protect cells and reduce oxidative damage caused by environmental factors and antimutagenic agents in various organisms [38].

In vitro assays assess the ability of a group of compounds to interact with a free neutral radical that possesses an unpaired electron in one of its orbitals [39]. Free radicals are highly reactive and unstable; when they seek stability by accepting electrons, they can initiate chain reactions that damage cellular structures, including membranes, lipids, and proteins [40]. Therefore, the peptides present in the fractions obtained from jellyfish collagen hydrolysates, which exhibit antioxidant properties, may help protect cells in organisms against oxidative stress by scavenging free radicals and enhancing natural antioxidant defenses. It has been shown that peptides derived from jellyfish collagen can increase superoxide dismutase levels [17], a key cellular antioxidant, and reduce reactive oxygen species [41].

The higher antioxidant and antimutagenic capacity of the fraction obtained after ultrafiltration through a membrane with a molecular weight cut-off below three kDa (F3) may be attributed to its exposure to a hydrophobic surface during the fractionation process [42]. This fraction could expose more amino acids to the surface, enhancing their interaction with free radicals, converting them into more stable products [42], and protecting cells against oxidative stress more effectively. The amino acid composition of the jellyfish collagen hydrolysates supports their observed antioxidant and antimutagenic activities. The relatively high content of glycine (23.1%), proline (9.0%), and hydroxyproline (4.0%) reflects the collagenous nature of the material. It contributes to the formation of low-molecular-weight peptides with flexible backbones and available electron-donating groups. The presence of tyrosine (3.1%) and methionine (1.5%) further enhances radical-scavenging capacity through hydrogen or electron donation, while the abundance of acidic residues (Asp + Glu = 22.0%) favors metal chelation and redox modulation. The high proportion of non-polar residues (48.0%) facilitates interaction with lipid radicals, supporting peroxyl radical quenching at hydrophobic interfaces. Moreover, the total essential to non-essential amino acid ratio (EAA/NEAA = 0.438) indicates a predominance of non-essential residues. Collectively, these features explain the strong antioxidant response and suggest a secondary antimutagenic effect through the reduction of reactive oxygen species and inhibition of oxidative DNA damage. Nevertheless, an amino acid profile of each fraction should be developed to confirm this. Moreover, the peptides’ efficiency in reacting with radicals as electron donors and preventing radical chain reactions is strongly affected by their amino acid sequence.

The structural characteristics of peptides are considered crucial to establishing their application as functional ingredients [43]. The antioxidant activity observed in both the hydrolysates and fractions F1, F2 and F3 could be related to peptide modifications, such as dehydration, glycation, and oxidation of aromatic rings, which alter their structure and, consequently, their bioactivity [44]. The distinctive antioxidant performance of fraction F3 could be attributed to the type of secondary structure formed by its peptides, as well as to the sequence of its amino acids [45]. It has been documented that peptides derived from hydrolyzed proteins can form both b-sheet and a-helix structures, with the b-structure being the most stable and capable of efficient electron donation [45]. Likewise, if amino acids such as aspartic acid, glutamic acid, or arginine are present in the peptide sequence, they can influence the interaction and the conformational properties of the peptides [46]. Further research is required to verify that the F3 fraction obtained under these study conditions contains peptides with amino acids identified in the aforementioned study. Involvement in these future studies is crucial to advancing the knowledge of bioactive compounds present in jellyfish collagen hydrolysate fractions.

Previous research has indicated that jellyfish collagen is chemically simple, which contributes to its versatile and adaptable tissue properties. As a result, it has been classified as collagen type 0 [47]. This type of collagen exhibits a chemical composition similar to that of various collagen classes [47] and shares numerous functions with more specialized collagens [48], including antioxidant properties [11], through the Peptide Ranker program and the ranks assigned to the probability of a specific peptide being bioactive [30]. The eight peptides identified with a substantial likelihood of bioactivity, containing fewer than 20 residues, with the presence of amino acids considered crucial for the antioxidant activities of peptides [16]: glycine, proline, leucine, alanine, phenylalanine, and valine.

Some of the peptides mentioned above could be present in the fractions obtained in this study. They may be responsible for the biological activities detected, whether antioxidant or antimutagenic. In this study, the specific molecular weight and sequences of the F3 peptides were not determined. This gap underscores the need for further research to determine the molecular weight distribution of the fraction and identify the F3 peptides that contribute to its bioactive properties. Understanding these sequences is crucial because they may play a vital role in the peptides’ mechanisms of action.

Furthermore, complementary in vitro cellular and in vivo animal studies are essential for fully validating the biological activity and therapeutic potential of these peptides.

## 4. Materials and Methods

### 4.1. Materials and Chemicals

Sixty-five jellyfish *Stomolophus sp. 2* specimens were collected in April 2023, during the seasonal blooming period from Kino Bay (28°43′ N/111°54′ W, 24–36 °C). The jellyfish organisms’ measures were 0.45 to 1.1 kg in weight and 12–17 cm in length. Specimens stored in an ice bed system were transported to the laboratory, and mesoglea was gathered from the organisms. Pepsin (4550 U/mg) and Alcalase^®^ (protease from *Bacillus licheniformis*, Type VIII, lyophilized powder, 7–15 U/mg) were purchased from Sigma-Aldrich (St. Louis, MO, USA). *Salmonella typhimurium* TA100 tester strains and the metabolic activation system S9 mix (Aroclor 1254-induced, Sprague–Dawley male rat liver in 0.154 M KCl solution) were purchased from Molecular Toxicology Inc. (Annapolis, MD, USA). All chemicals used were of analytical reagent grade and were purchased from Sigma-Aldrich. 

### 4.2. Collagen Extraction

The collagen extraction followed the procedure described previously [9]. Mesoglea jellyfish were cut into small pieces (100 g), and collagen was obtained with 0.1 M NaOH (1:5 *w*/*v*) and mechanically stirred for 24 h. After centrifugation (39,200× *g*, 4 °C, 90 min), the pellet was sequentially rinsed until the pH dropped to seven and freeze-dried. After that, it was treated with 0.5 M CH_3_COOH (1:5 *w*/*v*) and pepsin (10 mg/sample in 0.5 M CH_3_COOH; 1:5 *w*/*v*). At each step, after stirring for 24 h, the samples were centrifuged (39,200× *g*, 4 °C, 15 min). Finally, the samples dialyzed at 4 °C against water (cellulose membrane 50 kDa molecular weight cut-off) were lyophilized and stored at −80 °C for further analyses. The collagen present in the sample was determined by its hydroxyproline (Hyp) and protein content. Crude protein was quantified with a LECO FP-2000 Nitrogen Protein Analyzer (Method 9993.13, AOAC. 2000. “Official Methods of Analysis” 17th ed. Association of Analytical Chemists. Washington, DC, USA). Hyp was determined by high-performance liquid chromatography (RP-HPLC, Agilent Technologies Inc., Palo Alto, CA, USA) [49].

### 4.3. Preparation of Enzymatic Hydrolysates

Hydrolysates were produced using a commercial enzyme system (Alcalase) according to the previously described method [50], with some modifications. The pepsin-solubilized collagen (200 mg) was dissolved in a 100 mM sodium phosphate buffer of pH 7.5 (0.4 mg protein/mL). The beakers were placed in a 55 °C water bath under constant mixing for 5 h. The enzyme–substrate ratio (E/S) was 0.2% (*w*/*w*). The enzyme was inactivated by heating the sample to 95 °C for 15 min. The supernatants obtained after centrifugation (6000× *g*, 15 min) were considered to contain hydrolysates (JCH). The freeze-dried samples were stored at −80 °C for further assays.

### 4.4. Membrane Ultrafiltration

The JCH was dissolved in deionized water (1 mg/mL) and fractionated by ultrafiltration through an ultrafilter (Amicon Stirred Cell, Model 8200, Sigma-Aldrich, St. Louis, MO, USA) equipped with a 10 kDa membrane into one fraction: F1, composed of peptides with molecular weights > 10 kDa. The F3 was subjected to ultrafiltration using a 3 kDa membrane and fractionated into two fractions: F2, consisting of peptides from molecular weight > 3 kDa and <10 kDa, and F3, comprising peptides of molecular weight < 3 kDa. The fractionated temperature was 4 °C, and the pressure was 0.2 MPa. The collected fractions were freeze-dried and stored at −80 °C.

### 4.5. Analysis

#### 4.5.1. Degree of Hydrolysis (DH)

DH was established by analyzing free amino groups by reaction with the *O*-phthaldialdehyde (OPA) reagent [51]. Serine was used as a standard. The a, b, and htot values were 1.00, 0.40, and 8.6, respectively.

#### 4.5.2. Amino Acid Profile of Hydrolysates

The amino acid content of JCH was determined by reverse-phase high-performance liquid chromatography (Hewlett-Packard RP-HPLC, Agilent Technologies Inc., Santa Clara, CA, USA). Freeze-dried samples (100 mg) were hydrolyzed under reduced pressure (6 M HCl, 150 °C, 6 h). The hydrolysate residues, after centrifugation, were neutralized with 2 mL of 4 M NaOH and filtered through a cellulose acetate syringe filter unit (0.2 mm). After filtration, the samples were mixed with potassium borate buffer (pH 10.4) and *O*-phthaldialdehyde (1:1, *v*/*v*) and applied to the RP-HPLC system. The chromatogram recordings and integrations were performed using ChemStation software B.04.03 (Agilent Technologies). Fluorescence was measured at wavelengths of 330 nm (excitation) and 418 nm (emission) [52]. Analysis was performed in triplicate, and the results were expressed as g/100 g sample.

#### 4.5.3. Antioxidant Activity of Hydrolysates and Fractions

Three assays were conducted to evaluate the in vitro antioxidant activity of JCH and its fractions at a concentration of 0.75 mg/mL, a value established based on previously reported methodology and deemed significant for this research [9]. And the results were expressed as mmol TE (Trolox equivalent)/g.

The sample scavenging capacity to reduce the 2,2′-azino-bis-(3-ethylbenzothiazoline-6-sulfonic acid) radical cation (ABTS^●+^) was measured [53]. ABTS in water (7 mmol) was dissolved in 2.45 mmol potassium persulfate (dark room, 25 °C, 16 h) to produce the ABTS^●+^. Then, 20 μL aliquot samples were added to 980 μL of ABTS^●+^ diluted with methanol (Abs_734nm_ = 0.70), and the decrease in absorbance was measured at 734 nm using a 96-well microplate reader (Thermo Fisher Scientific Inc., Waltham, MA, USA).

The ferric reducing antioxidant power (FRAP) capacity of the samples was measured using a microplate reader, as described in previous studies [54]. The FRAP working solutions comprised 300 mM acetic acid–sodium acetate buffer (pH 3.6), 20 mM FeCl_3_•6 H_2_O, and 10 mM 2,4,6-tri(2-pyridyl)-s-triazine (TPTZ) in 40 mM HCl (10:1:1 ratio). The mixture of FRAP working solution (280 mL) and the sample (20 mL) was incubated at 25 °C in the dark for 30 min before its absorbance was recorded at 638 nm using a microplate reader.

Samples’ capacity to quench the oxygen radicals produced by the 2,2′-azobis(2-amidinopropane) (AAPH) was evaluated by the ORAC assay [55], which was the third chemical assay. The inhibition of fluorescence decay was assessed over 60 min at 37 °C at 485 nm (excitation) and 520 nm (emission) in a Cary Eclipse spectrophotometer (Agilent Technologies) after mixing 100 µL of the samples with a mixture of 75 mM phosphate buffer, pH 7.3 (1.7 mL), 0.36 M AAPH (100 µL), and 0.048 mM fluorescein (100 µL).

Furthermore, the concentration of the fraction (mg/mL) required to scavenge 50% (IC_50_) of ABTS+ was determined by plotting an inhibition curve from absorbance values obtained at various fraction concentrations.

#### 4.5.4. Antimutagenic Activity of Hydrolysates and Fractions

Salmonella/microsome assay [56] was employed to evaluate the antimutagenic potential of hydrolysates and fractions. Salmonella tester strain TA100 was used with bioactivation (S9, Aroclor 1254-induced, Sprague–Dawley male rat liver in 0.154 M KCl solution). Bacteria reproduction (2 × 10^9^ cells/mL, overnight culture, 37 °C) was developed in a nutrient broth. The mutagen employed was Aflatoxin B_1_ (with S9 mix) (500 ng/mL). The assay involved 100 μL of hydrolysates (0, 0.5, and 5 mg/mL) and fractions (0, 0.002, 0.02, 0.2, and 2.0 mg/mL) in test tubes. The dose gradients were established based on preliminary data and previously reported methodology [57]. Then, each tube was mixed with bacteriological agar containing histidine and biotin, bacterial culture (100 μL), and S9 mix (500 μL). 10% DMSO (100 μL) without AFB_1_ was used as a negative control. The mixture obtained was transferred to minimal agar plates. The plates were incubated (37 °C, 48 h), and each plate’s revertant bacterial colonies were counted. The inhibition percentage for mutagenic activity was calculated according to Equation (1).

(1) $$Percentage\;of\;inhibition\left(\%\right)=\left(1-\frac{\left[T-SR\right]}{\left[M-SR\right]}\right)\times 100$$

where *T* is the number of revertants in the presence of AFB_1_ and test sample plates, *M* is the positive control (number of revertants per plate in the mutagen without samples), and *SR* is the spontaneous revertant. A >40% was considered as strong antigenotoxicity, 25–40% moderate antigenotoxicity, and ≤25% no antigenotoxicity. Each dose was tested in triplicate.

#### 4.5.5. Genotoxicity Test of Hydrolysate and Fraction 3

Onions (*Allium cepa*) were allowed to germinate by immersion in distilled water and stored in the dark at 25 ± 2 °C. Onion roots about 5 cm long were used for testing. The onion roots were treated with hydrolysate and F3 samples at 50 and 100 ppm concentrations for 24 h. The control group was treated with distilled water. The root tips were dehydrated (45 min) in a 3:1 (*v*/*v*) ethanol–acetic acid solution, then fixed in 1 N hydrochloric acid (2 min, 60 °C). After that, the samples were stained with orcein for one minute and finally squashed. Mitotic cells were observed and counted under a microscope.

#### 4.5.6. Nano LC-MS/MS Analysis

The collagen sample was dissolved and identified with a nano LC-MS/MS platform (Ultimate 3000 nano-UHPLC system and Orbitrap Q Exactive HF mass spectrometer with Nanospray Flex Ion Source, Thermo Scientific, Poway, CA, USA) as previously reported [10]. The pepsin protein extracts were poured into a C18 SPE column (Thermo Scientific) with 0.1% formic acid to remove salts, and 1 mg of the sample was loaded into the Nanoflow UPLC. The MS/MS conditions were set as follows: scan range of 300–1650 *m*/*z* (resolution 60,000 at 200 *m*/*z*, 3 × 10^6^); operation in Top 20 mode (resolution 15,000 at 200 *m*/*z*; automatic gain control target 1 × 10^5^; maximum injection time 19 ms; normalized collision energy 28%; and an isolation window of 1.4 Th); and charge state exclusion parameters set to unassigned, 1, >6, and a dynamic exclusion of 30 s. Raw MS files were analyzed and searched against the jellyfish protein database based on the species of the samples using PEAKS Studio 8.5. The parameters were set as follows: the protein modifications were carbamidomethylation (fixed) and methionine oxidation (variable), and the enzyme specificity was pepsin. There were two maximum missed cleavages; precursor and ion mass tolerance were 10 ppm and 0.5 Da, respectively.

### 4.6. Statistical Analysis

The statistical design applied to the chemical characterization of jellyfish collagen hydrolysates and fractions was used to minimize replicate variation. Data (*n* = 3) obtained from the biological activities were subjected to the ANOVA method (*p* < 0.05) to investigate differences in variance using the SPSS^®^ program version 22.0 (SPSS Statistical Software, Inc., Chicago, IL, USA). Data were expressed as mean ± standard deviation out of three determinations. Significant differences (*p* < 0.05) between the results were identified using the Tukey test.

## 5. Conclusions

The hydrolysates derived from jellyfish collagen demonstrated notable in vitro antioxidant and antimutagenic properties, with no clastogenic effects. Following ultrafiltration, the antioxidant and antimutagenic activities of the hydrolysates were enhanced; however, clastogenicity remained unaffected. The highest bioactivity observed in fractions obtained after ultrafiltration through a membrane with a molecular weight cut-off below 3 kDa (F3) is likely due to peptides that aid in scavenging free radicals and blocking reactive oxygen species. These findings indicate that low-molecular-weight hydrolysate peptide fractions from jellyfish collagen warrant further investigation for purification and potential application as a bioactive food supplement. Moreover, given the large, underutilized biomass of this species in our region, this research represents an essential first step toward valorizing this marine resource. It provides a foundation for future, more advanced studies exploring its bioactive potential.

## Figures and Tables

**Table 1:** Antioxidant activity of mesoglea jellyfish *Stomolophus meleagris* collagen hydrolysates and their fractions obtained by ultrafiltration, measured by scavenging capacity of ABTS radical cation, to reduce Fe^3+^ to Fe^2+^ (FRAP), and scavenge oxygen-derived radicals (ORAC).

Antioxidant Activity (µmol TE/g *)			
Sample	ABTS	FRAP	ORAC
Hydrolysates	7920 ± 0.4 ^b^	6435 ± 2.1 ^b^	457 ± 4.3 ^b^
F1 (>10 kDa)	5763 ± 9.0 ^d^	1244 ± 1.1 ^d^	223 ± 2.5 ^d^
F2 (3–10 kDa)	6848 ± 3.1 ^c^	4345 ± 3.8 ^c^	324 ± 1.1 ^c^
F3 (<3 kDa)	8993 ± 5.2 ^a^	7622 ± 5.6 ^a^	599 ± 3.0 ^a^

* Values are the mean ± standard deviation of three independent determinations. Different letters (a–d) within the same column indicate significant differences indicate significant differences (*p* < 0.05) among the tested samples were assessed using one-way ANOVA and Tukey’s test.

**Table 2:** Effect of mesoglea jellyfish *Stomolophus meleagris* collagen hydrolysates and its fractions obtained by ultrafiltration on the mutagenicity induced by AFB_1_ using *Salmonella typhimurium* TA100 assay *.

Sample	Dose (mg/Plate)	Revertants/Plate	% Inhibition
Collagen	2	896 ± 3.0 ^f^	72.6 ± 0.3
	0.2	1444 ± 7.0 ^d^	55.8 ± 0.5
	0.02	2805 ± 3.0 ^b^	14.1 ± 0.1
	0.002	3245 ± 3.8 ^a^	0.6 ± 0.1
Hydrolysate	2	477 ± 6.0 ^n^	85.4 ± 1.2
	0.2	708 ± 7.0 ^k^	78.3 ± 0.9
	0.02	1323 ± 9.0 ^e^	59.5 ± 0.7
	0.002	1577 ± 5.2 ^c^	51.7 ± 0.3
F1 (>10 kDa)	2	464.9 ± 7.7 ^n^	85.8 ± 1.7
	0.2	598.5 ± 1.0 ^l^	81.7 ± 0.2
	0.02	725.2 ± 4.4 ^j^	77.8 ± 0.6
	0.002	822.9 ± 3.0 ^h^	74.8 ± 0.4
F2 (3–10 kDa)	2	401.3 ± 1.2 ^ñ^	87.7 ± 0.3
	0.2	590.6 ± 2.2 ^l^	81.9 ± 0.4
	0.02	706.2 ± 4.1 ^k^	78.4 ± 0.6
	0.002	814.6 ± 1.7 ^i^	75.1 ± 0.2
F3 (<3 kDa)	2	392.5 ± 2.9 ^ñ^	88.0 ± 0.7
	0.2	577.6 ± 3.1 ^m^	82.3 ± 0.5
	0.02	701.5 ± 1.4 ^k^	78.6 ± 0.2
	0.002	871.3 ± 3.0 ^g^	73.3 ± 0.3
Spontaneous Revertants		159.3 ± 12.9	
AFB_1_		3265.4 ± 26.2	

* Data are presented as the mean ± standard deviation of three replicates. Mean values followed by different letters (a–ñ) indicate significant differences (*p* < 0.05) among the tested doses and samples, determined by one-way ANOVA followed by Tukey’s post hoc test.

**Table 3:** Clastogenic effects of *Stomolophus* sp. 2 hydrolysates and fraction F3 (<3 kDa MW) on the mitotic cells of *Allium fistulosum*.

Sample	Mitotic Index * (%)	Abnormalities * (%)
Water	47.1 ± 0.80 ^c^	1.0 ± 0.01 ^d^
Sodium azide	43.2 ± 1.32 ^d^	47.26 ± 1.33 ^a^
Hydrolysates ^1^	53.9 ± 1.01 ^b^	2.01 ± 0.67 ^c^
Hydrolysates ^2^	55.7 ± 1.05 ^b^	3.08 ± 0.64 ^b^
F3 ^1^	58.2 ± 0.56 ^a^	1.02 ± 0.01 ^d^
F3 ^2^	59.8 ± 1.10 ^a^	1.16 ± 0.05 ^d^

^1^ Hydrolysates and F3 at 50 ppm. ^2^ Hydrolysates and F3 at 100 ppm. * Values represent the average of three repetitions ± SD. Different letters (a–d) indicate significant differences among samples (*p* < 0.05) determined by one-way ANOVA followed by Tukey’s post hoc test.

**Table 4:** Amino acid composition of jellyfish collagen hydrolysates obtained by Alcalase.

**Amino Acid**	g/100 g Protein *	**Antioxidant Relevance (Hydrophobicity)**	**Nutritional** **Classification**
Asp	9.0 ± 0.4	NH	NEAA
Glu	13.0 ± 0.6	NH	NEAA
Lys	3.6 ± 0.2	NH	EAA
Arg	10.0 ± 0.5	NH	EAA
Met	1.5 ± 0.1	H	EAA
Ser	7.1 ± 0.3	NH	NEAA
Thr	2.6 ± 0.1	NH	EAA
Gly	23.1 ± 1.2	H	NEAA
Ala	1.5 ± 0.1	H	NEAA
Val	2.7 ± 0.1	H	EAA
Ile	2.2 ± 0.1	H	EAA
Leu	5.9 ± 0.3	H	EAA
Tyr	3.1 ± 0.1	H	NEAA
Phe	2.1 ± 0.1	H	EAA
Pro	9.0 ± 0.4	H	NEAA
Hyp	4.0 ± 0.2	H	NEAA
Charged (+)	13.6 ± 0.7		
Charged (−)	22.0 ± 1.0		
Polar without charge	10.2 ± 0.4		
No polar	48.0 ± 2.4		
Aromatics	7.8 ± 0.3		
Pro + Hyp	13.0 ± 0.8		

* Values represent the average of three repetitions ± SD. H = hydrophobic, NH = non-hydrophobic. EAA = essential, NEAA = non-essential.

## Data Availability

The data presented in this study is available in the article. Further information is available upon request from the corresponding author.

## Abbreviations

The following abbreviations are used in this manuscript:

JCH	Jellyfish collagen hydrolysates
F1	Fraction molecular weight > 10 kDa
F2	Fraction 10 kDa > molecular weight > 3 kDA
F3	Fraction molecular weight > 3 kDa
ABTS	2,2′-azino-bis-(3-ethylbenzothiazoline-6-sulfonic-acid)
FRAP	Ferric reducing antioxidant power
AAPH	2,2′-azobis(2-methylpropionamidine) dihydrochloride
SET	Single-electron transfer
HAT	Hydrogen atom transfer
DNA	Deoxyribonucleic acid
DMSO	Dimethyl sulfoxide
LC-MS/MS	Liquid chromatography-tandem mass spectrometry
UHPLC	Ultra-high-performance liquid chromatography
ANOVA	Analysis of Variance
ORAC	Oxygen radical absorbance capacity

## References

[1] Cisneros-Mata M.A., Mangin T., Bone J., Rodriguez L., Smith S.L., Gaines S.D. (2019). Fisheries Governance in the Face of Climate Change: Assessment of Policy Reform Implications for Mexican Fisheries. PLoS ONE.

[2] Brotz L., Cisneros-Montemayor A.M., Cisneros-Mata M.Á. (2021). The Race for Jellyfish: Winners and Losers in Mexico’s Gulf of California. Mar. Policy.

[3] Gómez-Salinas L.C., López-Martínez J., Morandini A.C. (2021). The Young Stages of the Cannonball Jellyfish (*Stomolophus* sp. 2) from the Central Gulf of California (Mexico). Diversity.

[4] Riccio G., Martinez K.A., Martín J., Reyes F., D’Ambra I., Lauritano C. (2022). Jellyfish as an Alternative Source of Bioactive Antiproliferative Compounds. Mar. Drugs.

[5] Jarms G., Morandini A. (2019). World Atlas of Jellyfish.

[6] Banha T.N.S., Morandini A.C., Rosário R.P., Martinelli Filho J.E. (2020). *Scyphozoan jellyfish* (Cnidaria, Medusozoa) from Amazon Coast: Distribution, Temporal Variation and Length–Weight Relationship. J. Plankton Res..

[7] Sastré-Velásquez C.D., Rodríguez-Armenta C.M., Minjarez-Osorio C., Re-Vega E.D.L. (2022). Estado actual del conocimiento de la medusa bola de cañón (*Stomolophus meleagris*). Epistemus.

[8] Sagarhpa Favorece Gobierno de Sonora a Pescadores Con Asistencia Técnica y de Organización En El Golfo de Santa Clara. https://sagarhpa.sonora.gob.mx/acerca-de/acciones/favorece-gobierno-de-sonora-a-pescadores-con-asistencia-tecnica-y-de-organizacion-en-el-golfo-de-santa-clara.

[9] Villalba-Urquidy B.d.S., Torres-Arreola W., Toro-Sánchez C.L.D., Medina I., Burgos-Hernández A., Brauer J.M.E., Santacruz-Ortega H.d.C. (2025). Collagen Extracts from Blue Cannonball Jellyfish (*Stomolophus meleagris*): Antioxidant Properties, Chemical Structure, and Proteomic Identification. Ital. J. Food Sci..

[10] Esparza-Espinoza D.M., Del Carmen Santacruz-Ortega H., Plascencia-Jatomea M., Aubourg S.P., Salazar-Leyva J.A., Rodríguez-Felix F., Ezquerra-Brauer J.M. (2023). Chemical-Structural Identification of Crude Gelatin from Jellyfish (*Stomolophus meleagris*) and Evaluation of Its Potential Biological Activity. Fishes.

[11] Chiarelli P.G., Suh J.H., Pegg R.B., Chen J., Mis Solval K. (2023). The Emergence of Jellyfish Collagen: A Comprehensive Review on Research Progress, Industrial Applications, and Future Opportunities. Trends Food Sci. Technol..

[12] Lu J., Shi P., Cao Y., Shi B., Shen H., Zhao S., Gao Y., Chi H., Wang L., Shi Y. (2025). Isolation and Purification of Novel Antioxidant Peptides from Mussel (*Mytilus edulis*) Prepared by Marine Bacillus Velezensis Z-1 Protease. Mar. Drugs.

[13] Wang B., Li L., Chi C.-F., Ma J.-H., Luo H.-Y., Xu Y. (2013). Purification and Characterisation of a Novel Antioxidant Peptide Derived from Blue Mussel (*Mytilus edulis*) Protein Hydrolysate. Food Chem..

[14] Qiu Y.-T., Wang Y.-M., Yang X.-R., Zhao Y.-Q., Chi C.-F., Wang B. (2019). Gelatin and Antioxidant Peptides from Gelatin Hydrolysate of Skipjack Tuna (*Katsuwonus pelamis*) Scales: Preparation, Identification and Activity Evaluation. Mar. Drugs.

[15] Sheng Y., Qiu Y.-T., Wang Y.-M., Chi C.-F., Wang B. (2022). Novel Antioxidant Collagen Peptides of Siberian Sturgeon (Acipenserbaerii) Cartilages: The Preparation, Characterization, and Cytoprotection of H2O2-Damaged Human Umbilical Vein Endothelial Cells (HUVECs). Mar. Drugs.

[16] Zou T.-B., He T.-P., Li H.-B., Tang H.-W., Xia E.-Q. (2016). The Structure-Activity Relationship of the Antioxidant Peptides from Natural Proteins. Molecules.

[17] Teng L., Wang X., Yu H., Li R., Geng H., Xing R., Liu S., Li P. (2023). Jellyfish Peptide as an Alternative Source of Antioxidant. Antioxidants.

[18] Zhuang Y., Hou H., Zhao X., Zhang Z., Li B. (2009). Effects of Collagen and Collagen Hydrolysate from Jellyfish (*Rhopilema esculentum*) on Mice Skin Photoaging Induced by UV Irradiation. J. Food Sci..

[19] Upata M., Siriwoharn T., Makkhun S., Yarnpakdee S., Regenstein J.M., Wangtueai S. (2022). Tyrosinase Inhibitory and Antioxidant Activity of Enzymatic Protein Hydrolysate from Jellyfish (*Lobonema smithii*). Foods.

[20] Soufi-Kechaou E., Derouiniot-Chaplin M., Ben Amar R., Jaouen P., Berge J.-P. (2017). Recovery of Valuable Marine Compounds from Cuttlefish By-Product Hydrolysates: Combination of Enzyme Bioreactor and Membrane Technologies: Fractionation of Cuttlefish Protein Hydrolysates by Ultrafiltration: Impact on Peptidic Populations. Comptes Rendus Chim..

[21] Sila A., Bougatef A. (2016). Antioxidant Peptides from Marine By-Products: Isolation, Identification and Application in Food Systems. A Review. J. Funct. Foods.

[22] Raksha N., Halenova T., Vovk T., Kostyuk O., Synelnyk T., Andriichuk T., Maievska T., Savchuk O., Ostapchenko L. (2023). Anti-Obesity Effect of Collagen Peptides Obtained from *Diplulmaris antarctica*, a Jellyfish of the Antarctic Region. Croat. Med. J..

[23] Liu X., Zhang M., Jia A., Zhang Y., Zhu H., Zhang C., Sun Z., Liu C. (2013). Purification and Characterization of Angiotensin I Converting Enzyme Inhibitory Peptides from Jellyfish *Rhopilema esculentum*. Food Res. Int..

[24] Zhuang Y., Sun L., Li B. (2012). Production of the Angiotensin-I-Converting Enzyme (ACE)-Inhibitory Peptide from Hydrolysates of Jellyfish (*Rhopilema esculentum*) Collagen. Food Bioprocess. Technol..

[25] Owen R.W., Haubner R., Würtele G., Hull E., Spiegelhalder B., Bartsch H. (2004). Olives and Olive Oil in Cancer Prevention. Eur. J. Cancer Prev. Off. J. Eur. Cancer Prev. Organ. ECP.

[26] Wu H.-C., Pan B.S., Chang C.-L., Shiau C.-Y. (2005). Low-Molecular-Weight Peptides as Related to Antioxidant Properties of Chicken Essence. J. Food Drug Anal..

[27] Jurado J., Alejandre-Durán E., Pueyo C. (1993). Genetic Differences between the Standard Ames Tester Strains TA100 and TA98. Mutagenesis.

[28] Pandey H., Kumar V., Roy B.K. (2014). Assessment of Genotoxicity of Some Common Food Preservatives Using *Allium cepa* L. as a Test Plant. Toxicol. Rep..

[29] Guttuso P., Nogueira N., Gueroun S.K.M., Javidpour J., Canning-Clode J., Andrade C.A.P. (2025). Is Jellyfish a Suitable Ingredient for Aquafeed? A Comprehensive Review of Nutritional Potential and Limitation. Front. Mar. Sci..

[30] Mooney C., Haslam N.J., Pollastri G., Shields D.C. (2012). Towards the Improved Discovery and Design of Functional Peptides: Common Features of Diverse Classes Permit Generalized Prediction of Bioactivity. PLoS ONE.

[31] Xia Z., Miao J., Chen B., Guo J., Ou Y., Liang X., Yin Y., Tong X., Cao Y. (2022). Purification, Identification, and Antioxidative Mechanism of Three Novel Selenium-Enriched Oyster Antioxidant Peptides. Food Res. Int..

[32] Clauser K.R., Baker P., Burlingame A.L. (1999). Role of Accurate Mass Measurement (±10 Ppm) in Protein Identification Strategies Employing MS or MS/MS and Database Searching. Anal. Chem..

[33] Felician F.F., Yu R.-H., Li M.-Z., Li C.-J., Chen H.-Q., Jiang Y., Tang T., Qi W.-Y., Xu H.-M. (2019). The Wound Healing Potential of Collagen Peptides Derived from the Jellyfish *Rhopilema esculentum*. Chin. J. Traumatol..

[34] Derkus B., Arslan Y.E., Bayrac A.T., Kantarcioglu I., Emregul K.C., Emregul E. (2016). Development of a Novel Aptasensor Using Jellyfish Collagen as Matrix and Thrombin Detection in Blood Samples Obtained from Patients with Various Neurodisease. Sens. Actuators B Chem..

[35] Słoczyńska K., Powroźnik B., Pękala E., Waszkielewicz A.M. (2014). Antimutagenic Compounds and Their Possible Mechanisms of Action. J. Appl. Genet..

[36] Seo H.-S., Kim J.-S., Park M.-K., Seong N.-W., Kang G.-H., Kim S.-H., Kim J.-S., Kim S.-H., Kim J.-C., Moon C. (2024). Genotoxicity Evaluation of Collagen Peptide Derived from Skate (*Raja kenojei*) Skin: In Vitro and in Vivo Studies. Mol. Cell. Toxicol..

[37] Tian Y.-F., He C.-T., Chen Y.-T., Hsieh P.-S. (2013). Lipoic Acid Suppresses Portal Endotoxemia-Induced Steatohepatitis and Pancreatic Inflammation in Rats. World J. Gastroenterol..

[38] Purcarea C., Laslo V., Memete A.R., Agud E., Miere (Groza) F., Vicas S.I. (2022). Antigenotoxic and Antimutagenic Potentials of Proline in Allium Cepa Exposed to the Toxicity of Cadmium Antigenotoxic and Antimutagenic Potentials of Proline in Allium Cepa Exposed to the Toxicity of Cadmium. Agriculture.

[39] Tumilaar S.G., Hardianto A., Dohi H., Kurnia D. (2024). A Comprehensive Review of Free Radicals, Oxidative Stress, and Antioxidants: Overview, Clinical Applications, Global Perspectives, Future Directions, and Mechanisms of Antioxidant Activity of Flavonoid Compounds. J. Chem..

[40] Gulcin İ. (2025). Antioxidants: A Comprehensive Review. Arch. Toxicol..

[41] Batinic-Haberle I., Tovmasyan A., Roberts E.R.H., Vujaskovic Z., Leong K.W., Spasojevic I. (2014). SOD Therapeutics: Latest Insights into Their Structure-Activity Relationships and Impact on the Cellular Redox-Based Signaling Pathways. Antioxid. Redox Signal..

[42] Liu Y., Wan S., Liu J., Zou Y., Liao S. (2017). Antioxidant Activity and Stability Study of Peptides from Enzymatically Hydrolyzed Male Silkmoth. J. Food Process. Preserv..

[43] Zhu Y., Lao F., Pan X., Wu J. (2022). Food Protein-Derived Antioxidant Peptides: Molecular Mechanism, Stability and Bioavailability. Biomolecules.

[44] Udenigwe C.C., Fogliano V. (2017). Food Matrix Interaction and Bioavailability of Bioactive Peptides: Two Faces of the Same Coin?. J. Funct. Foods.

[45] Mirzaei M., Mirdamadi S., Safavi M., Soleymanzadeh N. (2020). The Stability of Antioxidant and ACE-Inhibitory Peptides as Influenced by Peptide Sequences. LWT.

[46] Yang S., Lin S., Ye H. (2022). Water Distribution and Moisture-Absorption in Egg-White Derived Peptides: Effects on Their Physicochemical, Conformational, Thermostable, and Self-Assembled Properties. Food Chem..

[47] Faruqui N., Williams D.S., Briones A., Kepiro I.E., Ravi J., Kwan T.O.C., Mearns-Spragg A., Ryadnov M.G. (2023). Extracellular Matrix Type 0: From Ancient Collagen Lineage to a Versatile Product Pipeline—JellaGel^TM^. Mater. Today Bio.

[48] Bowen A.J., Ekbom D.C., Hunter D., Voss S., Bartemes K., Mearns-Spragg A., Oldenburg M.S., San-Marina S. (2022). Larynx Proteomics after Jellyfish Collagen IL: Increased ECM/Collagen and Suppressed Inflammation. Laryngoscope Investig. Otolaryngol..

[49] Vázquez-Ortíz F.A., Morón-Fuenmayor O.E., González-Méndez N.F. (2004). Hydroxyproline Measurement by HPLC: Improved Method of Total Collagen Determination in Meat Samples. J. Liq. Chromatogr. Relat. Technol..

[50] Barzideh Z., Latiff A.A., Gan C.-Y., Abedin M.Z., Alias A.K. (2014). ACE Inhibitory and Antioxidant Activities of Collagen Hydrolysates from the Ribbon Jellyfish (*Chrysaora* sp.). Food Technol. Biotechnol..

[51] Nielsen P.M., Petersen D., Dambmann C. (2001). Improved Method for Determining Food Protein Degree of Hydrolysis. J. Food Sci..

[52] Vázquez-Ortiz F.A., Caire G., Higuera-Ciapara I., Hernández G. (1995). High Performance Liquid Chromatographic Determination of Free Amino Acids in Shrimp. J. Liq. Chromatogr..

[53] Re R., Pellegrini N., Proteggente A., Pannala A., Yang M., Rice-Evans C. (1999). Antioxidant Activity Applying an Improved ABTS Radical Cation Decolorization Assay. Free Radic. Biol. Med..

[54] Benzie I.F.F., Strain J.J. (1999). [2] Ferric Reducing/Antioxidant Power Assay: Direct Measure of Total Antioxidant Activity of Biological Fluids and Modified Version for Simultaneous Measurement of Total Antioxidant Power and Ascorbic Acid Concentration. Methods in Enzymology.

[55] Prior R.L., Hoang H., Gu L., Wu X., Bacchiocca M., Howard L., Hampsch-Woodill M., Huang D., Ou B., Jacob R. (2003). Assays for Hydrophilic and Lipophilic Antioxidant Capacity (Oxygen Radical Absorbance Capacity (ORACFL)) of Plasma and Other Biological and Food Samples. J. Agric. Food Chem..

[56] Maron D.M., Ames B.N. (1983). Revised Methods for the Salmonella Mutagenicity Test. Mutat. Res. Mutagen. Relat. Subj..

[57] Suárez-Jiménez G.M., Burgos-Hernández A., Torres-Arreola W., López-Saiz C.M., Velázquez Contreras C.A., Ezquerra-Brauer J.M. (2019). Bioactive Peptides from Collagen Hydrolysates from Squid (*Dosidicus gigas*) by-Products Fractionated by Ultrafiltration. Int. J. Food Sci. Technol..

